# 1-Acryloyl-2,6-bis­(4-chloro­phen­yl)-3,5-dimethyl­piperidin-4-one

**DOI:** 10.1107/S1600536809010472

**Published:** 2009-04-08

**Authors:** B. N. Lakshminarayana, J. Shashidhara Prasad, C. R. Gnanendra, M. A. Sridhar, Nagaraja Naik

**Affiliations:** a Department of Studies in Physics, Manasagangotri, University of Mysore, Mysore 570 006, India; b Department of Studies in Chemistry, Manasagangotri, University of Mysore, Mysore 570 006, India

## Abstract

In the crystal structure of the title compound, C_22_H_21_Cl_2_NO_2_, the piperidinone ring is in a boat conformation.

## Related literature

For the bioactivity of piperidin-4-ones, see: Jerom & Spencer (1988 ▶); Bochringer & Shochne (1961 ▶); Mobio *et al.* (1989 ▶). For ring-puckering analysis, see: Cremer & Pople (1975 ▶). For the synthesis, see: Baliah *et al.*, (1983 ▶). For a related structure, see: Ompraba *et al.* (2003 ▶).
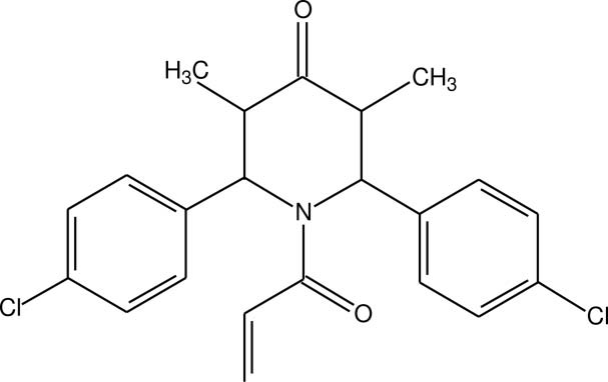


## Experimental

### 

#### Crystal data

C_22_H_21_Cl_2_NO_2_
*M* *_r_* = 402.30
Monoclinic, 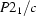

*a* = 10.2410 (8) Å
*b* = 19.5070 (11) Å
*c* = 10.9760 (9) Å
β = 112.567 (2)°
*V* = 2024.8 (3) Å^3^
*Z* = 4
Mo *K*α radiation
μ = 0.34 mm^−1^
*T* = 293 K
0.30 × 0.27 × 0.25 mm

#### Data collection

MacScience DIPLabo 32001 diffractometer
Absorption correction: none
6713 measured reflections
3542 independent reflections
2800 reflections with *I* > 2σ(*I*)
*R*_int_ = 0.025

#### Refinement

*R*[*F* ^2^ > 2σ(*F* ^2^)] = 0.044
*wR*(*F* ^2^) = 0.113
*S* = 1.03
3542 reflections
247 parameters
H-atom parameters constrained
Δρ_max_ = 0.20 e Å^−3^
Δρ_min_ = −0.25 e Å^−3^

### 

Data collection: *XPRESS* (MacScience, 2002 ▶); cell refinement: *SCALEPACK* (Otwinowski & Minor, 1997 ▶); data reduction: *DENZO* (Otwinowski & Minor, 1997 ▶) and *SCALEPACK*; program(s) used to solve structure: *SHELXS97* (Sheldrick, 2008 ▶); program(s) used to refine structure: *SHELXL97* (Sheldrick, 2008 ▶); molecular graphics: *PLATON* (Spek, 2009 ▶) and *ORTEPII* (Johnson, 1976 ▶); software used to prepare material for publication: *PLATON*.

## Supplementary Material

Crystal structure: contains datablocks global, I. DOI: 10.1107/S1600536809010472/pk2157sup1.cif

Structure factors: contains datablocks I. DOI: 10.1107/S1600536809010472/pk2157Isup2.hkl

Additional supplementary materials:  crystallographic information; 3D view; checkCIF report

## Figures and Tables

**Fig. 1.**
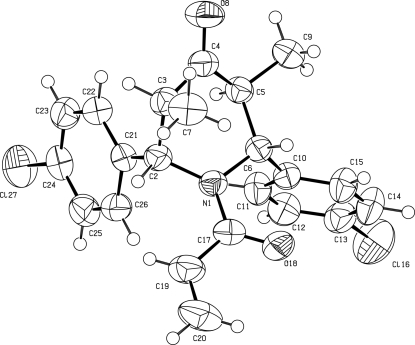
Crystal structure of the title compound with 50% probability displacement ellipsoids.

## supplementary crystallographic information

### Comment

The structural and therapeutic diversity of small heterocyclic molecules has attracted the attention of organic and medicinal chemists. Piperidin-4-ones are emerging prominently as pharmocologically important molecules because of their diverse bioactivities, such as anti-inflammatory (Jerom & Spencer, 1988), tranquilizers (Bochringer *et al.*, 1961) along with bactericidal, fungicidal and herbicidal activities (Mobio *et al.*, 1989). In view of the above, 1-acryloyl-2,6-bis(4-chlorophenyl)-3,5- dimethylpiperidin-4-one, was synthesized and its crystal structure is reported here.

A perspective view of the structure with the atomic numbering scheme is shown in Fig. 1. Ring-puckering analysis (Cremer & Pople, 1975) of the six-membered ring in the molecule indicates that the ring adopts a boat conformation, with a puckering amplitude Q=0.670 (2)Å, θ=85.71 (2)° and φ=72.45 (2)°. Atoms C2 and C6 deviate from the plane (Cremer & Pople, 1975) defined by the atoms N1/C/C3/C4/C5/C6   by -0.397 (2)Å and 0.240 (2)Å, respectively.  The piperidin ring in the molecule 1-acryloyl-2,6-bis(4-chlorophenyl)-3,5-dimethylpiperidin-4-one has a weighted average torsion angle of 34.35° (compare to 52.3° in 2,6-bis(4-chlorophenyl)-3-phenylpiperidin-4-one, Ompraba *et al.*, 2003). The substituent at C2 has an equatorial conformation as indicated by the dihedral angle of 86.87 (1)° between piperidin ring and phenyl ring and the substituent at C6 between the piperidin and phenyl ring has a dihedral angle of 77.81 (1)°. The methyl groups substituted at C3 and C5 are oriented in -syn-clinal and +anti-periplanar conformation as indicated by the torsion angle value of N1–C2–C3–C7, which is -65.3 (2)° and N1–C6–C5–C9, which is 173.51 (18)°.  The torsion angle value of -3.0 (3)° for C6–N1–C17–O18 indicates that O18 is oriented in a -syn-periplanar conformation.

### Experimental

To a well stirred solution of 2,6-bis(4-chlorophenyl)-3,5-dimethylpiperidin-4-one (Baliah *et al.*, 1983) (5 mmol) and triethylamine(5 mmol) in 30 ml of benzene, 3-chloropropanoyl chloride (5 mmol) in 20 ml benzene was added drop wise through a funnel for about an hour. The resulting mixture was stirred for about 4 hours under ambient conditions. After the completion of the reaction the mixture was quenched in cold water and the organic layer was extracted into ethyl acetate, washed with 5% sodium bicarbonate solution and dried over anhydrous sodium sulphate. This upon evaporation and recrystallization in alcohol yielded 2,6-bis(4-chlorophenyl)-1-(3-chloropropanoyl)-3,5-dimethylpiperidin-4-one. The crystals were dissolved in ethanol (60 ml), refluxed for half an hour and allowed to crystallize by slow evaporation of ethanol.

### Refinement

H atoms were placed at idealised positions and allowed to ride on their parent atoms with C–H distances in the range 0.93–0.98 Å; U_iso_(H) set to either 1.2U_eq_ or 1.5U_eq_ of the carrier atom.

### Crystal data

**Table:**

C_22_H_21_Cl_2_NO_2_	*F*(000) = 840
*M**_r_* = 402.30	*D*_x_ = 1.320 Mg m^−^^3^
Monoclinic, *P*2_1_/*c*	Mo *K*α radiation, λ = 0.71073 Å
Hall symbol: -P 2ybc	Cell parameters from 6713 reflections
*a* = 10.2410 (8) Å	θ = 3.0–25.0°
*b* = 19.5070 (11) Å	µ = 0.34 mm^−^^1^
*c* = 10.9760 (9) Å	*T* = 293 K
β = 112.567 (2)°	Block, colourless
*V* = 2024.8 (3) Å^3^	0.30 × 0.27 × 0.25 mm
*Z* = 4	

### Data collection

**Table:**

MacScience DIPLabo 32001 diffractometer	2800 reflections with *I* > 2σ(*I*)
Radiation source: fine-focus sealed tube	*R*_int_ = 0.025
graphite	θ_max_ = 25.0°, θ_min_ = 3.0°
Detector resolution: 10.0 pixels mm^-1^	*h* = −12→12
ω scans	*k* = −23→23
6713 measured reflections	*l* = −13→13
3542 independent reflections	

### Refinement

**Table:**

Refinement on *F*^2^	Secondary atom site location: difference Fourier map
Least-squares matrix: full	Hydrogen site location: inferred from neighbouring sites
*R*[*F*^2^ > 2σ(*F*^2^)] = 0.044	H-atom parameters constrained
*wR*(*F*^2^) = 0.113	*w* = 1/[σ^2^(*F*_o_^2^) + (0.0437*P*)^2^ + 0.7531*P*] where *P* = (*F*_o_^2^ + 2*F*_c_^2^)/3
*S* = 1.03	(Δ/σ)_max_ = 0.001
3542 reflections	Δρ_max_ = 0.20 e Å^−^^3^
247 parameters	Δρ_min_ = −0.25 e Å^−^^3^
0 restraints	Extinction correction: *SHELXL97* (Sheldrick, 2008), Fc^*^=kFc[1+0.001xFc^2^λ^3^/sin(2θ)]^-1/4^
Primary atom site location: structure-invariant direct methods	Extinction coefficient: 0.0072 (15)

### Special details

**Table:**

Geometry. Bond distances, angles *etc*. have been calculated using the rounded fractional coordinates. All su's are estimated from the variances of the (full) variance-covariance matrix. The cell e.s.d.'s are taken into account in the estimation of distances, angles and torsion angles
Refinement. Refinement of *F*^2^ against ALL reflections. The weighted *R*-factor *wR* and goodness of fit *S* are based on *F*^2^, conventional *R*-factors *R* are based on *F*, with *F* set to zero for negative *F*^2^. The threshold expression of *F*^2^ > σ(*F*^2^) is used only for calculating *R*-factors(gt) *etc*. and is not relevant to the choice of reflections for refinement. *R*-factors based on *F*^2^ are statistically about twice as large as those based on *F*, and *R*- factors based on ALL data will be even larger.

### Fractional atomic coordinates and isotropic or equivalent isotropic displacement parameters (Å^2^)

**Table:**

	*x*	*y*	*z*	*U*_iso_*/*U*_eq_	
Cl16	0.26934 (10)	0.75475 (4)	0.51117 (9)	0.1061 (4)	
Cl27	0.37661 (8)	0.35272 (4)	0.88810 (6)	0.0863 (3)	
O8	0.44186 (17)	0.35510 (9)	0.20478 (18)	0.0739 (7)	
O18	−0.06985 (15)	0.51370 (8)	0.12166 (15)	0.0631 (6)	
N1	0.09295 (16)	0.43755 (8)	0.24406 (16)	0.0460 (5)	
C2	0.1348 (2)	0.36689 (10)	0.2923 (2)	0.0487 (6)	
C3	0.2196 (2)	0.33554 (11)	0.2177 (2)	0.0524 (7)	
C4	0.3467 (2)	0.37817 (11)	0.2317 (2)	0.0507 (7)	
C5	0.3488 (2)	0.45090 (10)	0.2808 (2)	0.0475 (6)	
C6	0.20081 (19)	0.48388 (10)	0.23015 (19)	0.0449 (6)	
C7	0.1287 (2)	0.32667 (14)	0.0708 (2)	0.0694 (8)	
C9	0.4536 (2)	0.49632 (13)	0.2509 (3)	0.0714 (9)	
C10	0.2080 (2)	0.55179 (10)	0.29977 (19)	0.0466 (6)	
C11	0.2401 (2)	0.55513 (11)	0.4340 (2)	0.0571 (7)	
C12	0.2581 (2)	0.61714 (12)	0.4992 (2)	0.0627 (8)	
C13	0.2426 (2)	0.67645 (11)	0.4290 (3)	0.0631 (8)	
C14	0.2087 (3)	0.67519 (12)	0.2960 (3)	0.0735 (10)	
C15	0.1915 (3)	0.61283 (12)	0.2318 (2)	0.0636 (8)	
C17	−0.0434 (2)	0.45859 (11)	0.1803 (2)	0.0512 (7)	
C19	−0.1601 (2)	0.41532 (13)	0.1856 (3)	0.0693 (9)	
C20	−0.2902 (3)	0.43327 (19)	0.1270 (3)	0.0892 (13)	
C21	0.2020 (2)	0.36349 (10)	0.4431 (2)	0.0490 (6)	
C22	0.3290 (2)	0.33092 (11)	0.5125 (2)	0.0569 (8)	
C23	0.3825 (2)	0.32739 (12)	0.6491 (2)	0.0628 (8)	
C24	0.3089 (3)	0.35647 (11)	0.7166 (2)	0.0594 (8)	
C25	0.1816 (3)	0.38854 (13)	0.6510 (2)	0.0658 (9)	
C26	0.1296 (2)	0.39179 (12)	0.5151 (2)	0.0600 (8)	
H2	0.04690	0.34050	0.26590	0.0580*	
H3	0.25260	0.29010	0.25510	0.0630*	
H5	0.38110	0.44830	0.37710	0.0570*	
H6	0.17350	0.49350	0.13600	0.0540*	
H7A	0.09920	0.37090	0.03120	0.1040*	
H7B	0.04700	0.29960	0.06080	0.1040*	
H7C	0.18290	0.30400	0.02820	0.1040*	
H9A	0.54450	0.47450	0.28190	0.1070*	
H9B	0.46110	0.53970	0.29450	0.1070*	
H9C	0.42130	0.50350	0.15740	0.1070*	
H11	0.24990	0.51470	0.48150	0.0690*	
H12	0.28050	0.61850	0.58970	0.0750*	
H14	0.19730	0.71590	0.24900	0.0880*	
H15	0.16830	0.61200	0.14120	0.0760*	
H19	−0.13880	0.37430	0.23230	0.0830*	
H20A	−0.31280	0.47420	0.08010	0.1070*	
H20B	−0.36180	0.40530	0.13170	0.1070*	
H22	0.37920	0.31110	0.46670	0.0680*	
H23	0.46800	0.30540	0.69450	0.0750*	
H25	0.13140	0.40770	0.69740	0.0790*	
H26	0.04360	0.41360	0.47040	0.0720*	

### Atomic displacement parameters (Å^2^)

**Table:**

	*U*^11^	*U*^22^	*U*^33^	*U*^12^	*U*^13^	*U*^23^
Cl16	0.1353 (7)	0.0604 (4)	0.1182 (7)	0.0015 (4)	0.0437 (6)	−0.0258 (4)
Cl27	0.1033 (6)	0.0961 (5)	0.0559 (4)	−0.0045 (4)	0.0265 (4)	0.0072 (3)
O8	0.0597 (10)	0.0782 (11)	0.0950 (13)	0.0049 (8)	0.0422 (9)	−0.0148 (9)
O18	0.0510 (9)	0.0705 (10)	0.0633 (10)	0.0131 (7)	0.0168 (7)	0.0124 (8)
N1	0.0388 (9)	0.0478 (9)	0.0506 (9)	0.0018 (7)	0.0162 (7)	0.0009 (7)
C2	0.0431 (11)	0.0457 (10)	0.0580 (12)	−0.0012 (8)	0.0202 (9)	−0.0002 (9)
C3	0.0541 (12)	0.0481 (11)	0.0566 (12)	0.0027 (9)	0.0229 (10)	−0.0034 (9)
C4	0.0445 (11)	0.0594 (12)	0.0490 (11)	0.0057 (9)	0.0187 (9)	0.0014 (9)
C5	0.0430 (11)	0.0511 (11)	0.0494 (11)	−0.0004 (8)	0.0187 (9)	0.0013 (9)
C6	0.0415 (10)	0.0483 (11)	0.0441 (10)	0.0007 (8)	0.0155 (8)	0.0032 (8)
C7	0.0609 (14)	0.0813 (16)	0.0628 (14)	−0.0048 (12)	0.0203 (12)	−0.0189 (12)
C9	0.0537 (14)	0.0697 (15)	0.0981 (19)	−0.0016 (11)	0.0374 (13)	0.0114 (14)
C10	0.0421 (10)	0.0479 (11)	0.0482 (11)	0.0011 (8)	0.0155 (9)	0.0036 (9)
C11	0.0695 (14)	0.0515 (12)	0.0513 (12)	−0.0003 (10)	0.0242 (11)	0.0054 (9)
C12	0.0714 (15)	0.0634 (14)	0.0540 (13)	0.0008 (11)	0.0247 (11)	−0.0052 (11)
C13	0.0644 (14)	0.0496 (12)	0.0752 (16)	0.0025 (10)	0.0268 (12)	−0.0054 (11)
C14	0.0946 (19)	0.0473 (13)	0.0759 (17)	0.0084 (12)	0.0297 (14)	0.0120 (12)
C15	0.0780 (16)	0.0575 (13)	0.0522 (13)	0.0060 (11)	0.0214 (11)	0.0102 (10)
C17	0.0438 (11)	0.0614 (13)	0.0490 (11)	0.0031 (9)	0.0184 (9)	−0.0062 (10)
C19	0.0463 (13)	0.0713 (15)	0.0906 (18)	−0.0025 (11)	0.0267 (12)	−0.0075 (13)
C20	0.0502 (15)	0.136 (3)	0.0838 (19)	−0.0066 (15)	0.0283 (14)	−0.0043 (18)
C21	0.0496 (11)	0.0448 (10)	0.0566 (12)	−0.0021 (9)	0.0248 (10)	0.0051 (9)
C22	0.0548 (13)	0.0563 (12)	0.0637 (14)	0.0058 (10)	0.0273 (11)	0.0052 (11)
C23	0.0582 (13)	0.0618 (13)	0.0651 (14)	0.0022 (11)	0.0199 (11)	0.0116 (11)
C24	0.0695 (15)	0.0561 (12)	0.0547 (13)	−0.0089 (11)	0.0261 (11)	0.0063 (10)
C25	0.0763 (16)	0.0662 (15)	0.0659 (15)	0.0037 (12)	0.0396 (13)	0.0048 (12)
C26	0.0577 (13)	0.0656 (14)	0.0630 (14)	0.0114 (10)	0.0303 (11)	0.0086 (11)

### Geometric parameters (Å, °)

**Table:**

Cl16—C13	1.741 (3)	C22—C23	1.387 (3)
Cl27—C24	1.740 (2)	C23—C24	1.367 (4)
O8—C4	1.209 (3)	C24—C25	1.374 (4)
O18—C17	1.229 (3)	C25—C26	1.380 (3)
N1—C2	1.480 (3)	C2—H2	0.9800
N1—C6	1.480 (3)	C3—H3	0.9800
N1—C17	1.363 (3)	C5—H5	0.9800
C2—C3	1.531 (3)	C6—H6	0.9800
C2—C21	1.531 (3)	C7—H7A	0.9600
C3—C4	1.502 (3)	C7—H7B	0.9600
C3—C7	1.531 (3)	C7—H7C	0.9600
C4—C5	1.515 (3)	C9—H9A	0.9600
C5—C6	1.541 (3)	C9—H9B	0.9600
C5—C9	1.522 (3)	C9—H9C	0.9600
C6—C10	1.517 (3)	C11—H11	0.9300
C10—C11	1.383 (3)	C12—H12	0.9300
C10—C15	1.381 (3)	C14—H14	0.9300
C11—C12	1.381 (3)	C15—H15	0.9300
C12—C13	1.365 (3)	C19—H19	0.9300
C13—C14	1.365 (4)	C20—H20A	0.9300
C14—C15	1.383 (3)	C20—H20B	0.9300
C17—C19	1.482 (3)	C22—H22	0.9300
C19—C20	1.286 (4)	C23—H23	0.9300
C21—C22	1.384 (3)	C25—H25	0.9300
C21—C26	1.389 (3)	C26—H26	0.9300
			
C2—N1—C6	118.73 (16)	C21—C2—H2	106.00
C2—N1—C17	124.27 (17)	C2—C3—H3	108.00
C6—N1—C17	114.92 (16)	C4—C3—H3	108.00
N1—C2—C3	109.06 (16)	C7—C3—H3	108.00
N1—C2—C21	112.06 (16)	C4—C5—H5	107.00
C3—C2—C21	116.68 (18)	C6—C5—H5	107.00
C2—C3—C4	111.79 (17)	C9—C5—H5	107.00
C2—C3—C7	111.59 (18)	N1—C6—H6	108.00
C4—C3—C7	108.85 (17)	C5—C6—H6	108.00
O8—C4—C3	121.2 (2)	C10—C6—H6	108.00
O8—C4—C5	122.2 (2)	C3—C7—H7A	109.00
C3—C4—C5	116.64 (18)	C3—C7—H7B	109.00
C4—C5—C6	112.65 (17)	C3—C7—H7C	109.00
C4—C5—C9	112.55 (19)	H7A—C7—H7B	109.00
C6—C5—C9	110.90 (17)	H7A—C7—H7C	109.00
N1—C6—C5	112.19 (16)	H7B—C7—H7C	109.00
N1—C6—C10	112.04 (17)	C5—C9—H9A	110.00
C5—C6—C10	109.39 (16)	C5—C9—H9B	109.00
C6—C10—C11	121.58 (18)	C5—C9—H9C	109.00
C6—C10—C15	120.58 (18)	H9A—C9—H9B	109.00
C11—C10—C15	117.71 (19)	H9A—C9—H9C	109.00
C10—C11—C12	121.55 (19)	H9B—C9—H9C	109.00
C11—C12—C13	119.1 (2)	C10—C11—H11	119.00
Cl16—C13—C12	119.3 (2)	C12—C11—H11	119.00
Cl16—C13—C14	119.65 (18)	C11—C12—H12	120.00
C12—C13—C14	121.0 (2)	C13—C12—H12	120.00
C13—C14—C15	119.4 (2)	C13—C14—H14	120.00
C10—C15—C14	121.2 (2)	C15—C14—H14	120.00
O18—C17—N1	120.6 (2)	C10—C15—H15	119.00
O18—C17—C19	120.0 (2)	C14—C15—H15	119.00
N1—C17—C19	119.40 (19)	C17—C19—H19	119.00
C17—C19—C20	121.4 (3)	C20—C19—H19	119.00
C2—C21—C22	123.55 (19)	C19—C20—H20A	120.00
C2—C21—C26	118.78 (19)	C19—C20—H20B	120.00
C22—C21—C26	117.61 (19)	H20A—C20—H20B	120.00
C21—C22—C23	121.1 (2)	C21—C22—H22	119.00
C22—C23—C24	119.6 (2)	C23—C22—H22	120.00
Cl27—C24—C23	119.7 (2)	C22—C23—H23	120.00
Cl27—C24—C25	119.3 (2)	C24—C23—H23	120.00
C23—C24—C25	121.0 (2)	C24—C25—H25	121.00
C24—C25—C26	118.8 (2)	C26—C25—H25	121.00
C21—C26—C25	121.9 (2)	C21—C26—H26	119.00
N1—C2—H2	106.00	C25—C26—H26	119.00
C3—C2—H2	106.00		
			
C6—N1—C2—C3	−46.8 (2)	C4—C5—C6—C10	171.32 (17)
C6—N1—C2—C21	83.9 (2)	C9—C5—C6—N1	173.51 (18)
C17—N1—C2—C3	115.9 (2)	C9—C5—C6—C10	−61.5 (2)
C17—N1—C2—C21	−113.4 (2)	N1—C6—C10—C11	59.9 (3)
C2—N1—C6—C5	−4.6 (2)	N1—C6—C10—C15	−124.3 (2)
C2—N1—C6—C10	−128.09 (18)	C5—C6—C10—C11	−65.2 (3)
C17—N1—C6—C5	−168.89 (17)	C5—C6—C10—C15	110.6 (2)
C17—N1—C6—C10	67.6 (2)	C6—C10—C11—C12	174.6 (2)
C2—N1—C17—O18	−166.34 (19)	C15—C10—C11—C12	−1.3 (3)
C2—N1—C17—C19	15.5 (3)	C6—C10—C15—C14	−174.9 (3)
C6—N1—C17—O18	−3.0 (3)	C11—C10—C15—C14	1.0 (4)
C6—N1—C17—C19	178.84 (19)	C10—C11—C12—C13	0.6 (3)
N1—C2—C3—C4	56.9 (2)	C11—C12—C13—Cl16	−178.46 (18)
N1—C2—C3—C7	−65.3 (2)	C11—C12—C13—C14	0.5 (4)
C21—C2—C3—C4	−71.3 (2)	Cl16—C13—C14—C15	178.2 (2)
C21—C2—C3—C7	166.51 (18)	C12—C13—C14—C15	−0.8 (4)
N1—C2—C21—C22	−130.3 (2)	C13—C14—C15—C10	0.0 (5)
N1—C2—C21—C26	52.7 (3)	O18—C17—C19—C20	1.8 (4)
C3—C2—C21—C22	−3.5 (3)	N1—C17—C19—C20	179.9 (3)
C3—C2—C21—C26	179.44 (19)	C2—C21—C22—C23	−177.7 (2)
C2—C3—C4—O8	163.9 (2)	C26—C21—C22—C23	−0.7 (3)
C2—C3—C4—C5	−16.1 (2)	C2—C21—C26—C25	177.8 (2)
C7—C3—C4—O8	−72.4 (3)	C22—C21—C26—C25	0.6 (3)
C7—C3—C4—C5	107.6 (2)	C21—C22—C23—C24	0.0 (3)
O8—C4—C5—C6	144.5 (2)	C22—C23—C24—Cl27	−179.46 (18)
O8—C4—C5—C9	18.3 (3)	C22—C23—C24—C25	0.8 (4)
C3—C4—C5—C6	−35.4 (2)	Cl27—C24—C25—C26	179.38 (19)
C3—C4—C5—C9	−161.70 (19)	C23—C24—C25—C26	−0.8 (4)
C4—C5—C6—N1	46.3 (2)	C24—C25—C26—C21	0.2 (4)
